# Supplementary material for: Comprehensive Evaluation of the Nutritional Properties of Different Germplasms of Polygonatum cyrtonema Hua
Source: Foods. 2024 Mar 7;13(6):815. doi: 10.3390/foods13060815 (PMC10968880; doi:10.3390/foods13060815)
Supplement: Supplementary file 1 [file foods-13-00815-s001.zip › foods-2857790-supplementary.pdf]

Table S1. Specific information on the provenance of the five *P. cyrtonema*.

**Sample name**	**Specific provenance**	**Latitude （N）**	**Longitude （E）**	**Average Temperatures (℃)**	**Annual Rainfall (mm)**
Huanggang	Huanggag, Hubei	30°31′24"	114°55′37"	17.41	1536.10
Chizhou	Chizhou, Anhui	30°40′16"	117°30′19"	16.75	1431.59
Qingyang	Qingyang, Anhui	30°34′15"	117°48′22"	16.53	1485.03
Yueyang1	Yueyang, Hunan	29°32′42"	113°32′17"	17.51	1349.47
Yueyang2	Yueyang, Hunan	29°32′52"	113°32′08"	17.51	1349.47

Note: Data on average temperature and annual rainfall in 1950-2023 were obtained from the National Centers for Environmental Information (https://www.ngdc.noaa.gov/) (accessed on 3 December 2023).
